# Recent Advances in Therapeutic Strategies to Improve Colorectal Cancer Treatment

**DOI:** 10.3390/cancers16051029

**Published:** 2024-03-02

**Authors:** William H. Gmeiner

**Affiliations:** Department of Cancer Biology, Wake Forest University School of Medicine, Winston-Salem, NC 27157, USA; bgmeiner@wakehealth.edu

**Keywords:** colorectal cancer, chemotherapy precision oncology, fluoropyrimidine

## Abstract

**Simple Summary:**

Colorectal cancer is a leading cause of cancer mortality, and its incidence is rapidly increasing worldwide. Colorectal cancer is considered a single entity based on anatomical localization; however, it consists of multiple disease sub-types that are regulated by distinct cell signaling programs that present different risks for disease progression. It is important to develop chemotherapy approaches that target specific vulnerabilities for each major colorectal cancer sub-type. Since alternative pathways may become activated in response to the inhibition of a primary signaling pathway, the implementation of multi-component, comprehensive strategies that are founded in cancer biology is required for successful treatment.

**Abstract:**

Colorectal cancer (CRC) is the second-leading cause of cancer-related mortality worldwide. CRC mortality results almost exclusively from metastatic disease (mCRC) for which systemic chemotherapy is often a preferred therapeutic option. Biomarker-based stratification of mCRC enables the use of precision therapy based on individual tumor mutational profiles. Activating mutations in the RAS/RAF/MAPK pathway downstream of EGFR signaling have, until recently, limited the use of EGFR-targeted therapies for mCRC; however, the development of anti-RAS and anti-RAF therapies together with improved strategies to limit compensatory signaling pathways is resulting in improved survival rates in several highly lethal mCRC sub-types (e.g., BRAF-mutant). The use of fluoropyrimidine (FP)-based chemotherapy regimens to treat mCRC continues to evolve contributing to improved long-term survival. Future advances in chemotherapy for mCRC will need to position development relative to the advances made in precision oncology.

## 1. Introduction

Colorectal cancer (CRC) is newly diagnosed in >1.9 million individuals each year worldwide and is considered a single entity based on anatomical localization in the large intestine affecting the colon or rectum. However, CRC is a heterogeneous collection of cancer sub-types differentiated based on precise anatomical location (e.g., left-sided, colon) and cellular properties including microsatellite instability (MSI) and RAS mutation status that direct the course of treatment, particularly for metastatic CRC (mCRC). CRC collectively is a leading cause of cancer-related mortality worldwide resulting in >930,000 deaths [1]. By 2040, CRC-related deaths are projected to increase to >3.2 million new cases per year causing >1.6 million new deaths. Recent trends in the U.S. counter the rapid global increase in CRC incidence and mortality with a 1.2% decline reported in CRC incidence according to U.S. cancer statistics. However, with 153,000 new cases annually and 52,500 deaths, CRC is the third-leading cause of cancer-related deaths among men and women combined in the U.S. [2]. CRC is also increasingly being diagnosed in younger individuals [3] with >20% of new CRC cases now diagnosed in individuals 55 years or younger. CRC is also a malignancy associated with serious health disparities related to both race [4] and gender [5]. While survival rates in the U.S. are among the highest in the world with ~65% of CRC patients (all stages and sites combined) surviving 5 years, mCRC remains highly lethal with <11% of mCRC patients surviving 5 years [6].

The dismal 5-year survival rate for mCRC represents a substantial improvement relative to the survival rate before the introduction and optimization of fluoropyrimidine (FP) chemotherapy [7]. 5-Fluororuracil (5-FU) was introduced into the clinic >60 years ago, and its use in the adjuvant setting in combination with folinic acid (leucovorin (LV)) was shown to decrease the risk for disease recurrence for patients with primary colon cancer [8,9]. 5-FU/LV is useful in palliative therapy for mCRC, but the development of effective doublet combination therapy regimens, including FOLFOX (folinic acid (leucovorin), 5-FU, oxaliplatin) and FOLFIRI (leucovorin, 5-FU, irinotecan) [10], resulted in significant survival advantages. Recent trends in survival in mCRC using FP doublet therapies are encouraging, and the median overall survival (OS) for mCRC now exceeds 32 months, an improvement from 22.6 months in 2012 [11]. Numerous factors undoubtedly contribute to improved survival in mCRC; however, precision targeting based on the specific mutational profiles of individual CRC tumors is playing an important role [12]. Multiple clinical trials reported in recent years have noted significantly improved survival using precision therapeutic approaches that target some of the deadliest mCRC sub-types, including BRAF^V600E^ mutations [13]. Improved survival using targeted therapies has resulted from not only the discovery of new drugs that target actionable driver mutations but also the elucidation of the mechanisms for compensatory signaling pathways that are activated in response to target inhibition [14]. Improvements in precision oncology for mCRC are augmenting gains achieved through the introduction of immunotherapy [15] and improved surgical techniques that collectively are contributing toward improved long-term survival for distinct sub-types of mCRC [16].

In this review, we first address biomarker-based stratification for personalized therapy of mCRC with an emphasis on recent developments in anti-EGFR, anti-RAS, and anti-BRAF therapies and the use of anti-VEGF therapy in front-line and second-line settings. We then address chemotherapeutic options in the third-line setting of mCRC and the development of novel targeted therapies with the potential to further improve outcomes in mCRC.

## 2. Biomarker-Based Stratification of mCRC

Approaches for precision targeting of mCRC sub-types derive from an understanding of colorectal carcinogenesis initiated in the pioneering work of Fearon and Vogelstein [17]. The initiating event for adenomas is mutation in APC, a component of the destruction complex that degrades β-catenin, while the initiating event for serrated polyps involves an activating mutation in the BRAF oncogene [18]. The adenoma-to-carcinoma progression proceeds via the chromosomal instability (CIN) pathway in ~65–70% of sporadic CRC with the MSI pathway (~15%) and serrated neoplasia (~15%) alternative pathways of CRC carcinogenesis [18]. CIN involves an accelerated rate in either gain or loss of large parts or entire chromosomes or chromosomal rearrangements [19] with 17 sub-types of CIN identified in a pan-cancer analysis [20]. While CRC developing via the CIN pathways is characterized by defects in chromosomal segregation, tumors developing via this mechanism are not hypermutated, and current immunotherapy is generally not effective for these tumors.

**Figure 1:**
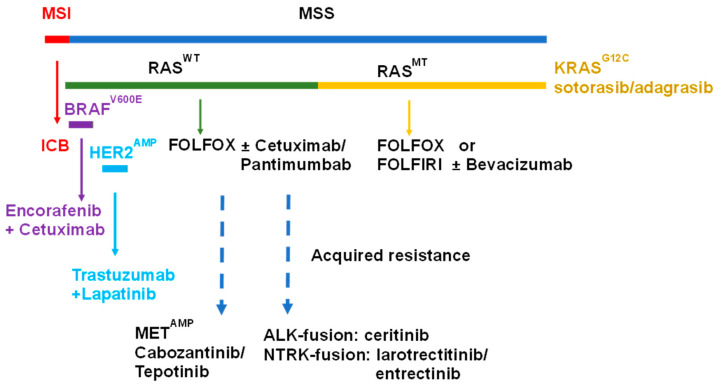
Biomarker-based stratification for front-line treatment of mCRC. ESMO and NCCN guidelines recommend MSI/MSS testing and comprehensive RAS and BRAF mutation status. Most mCRC is MSS (~95%) and treated with a combination of chemotherapy and targeted therapy. MSI (~5%) is treated with immune checkpoint blockade (ICB) immunotherapy. About 40% of mCRC expresses a mutant RAS protein (KRAS or NRAS), and front-line treatment frequently consists of either FOLFOX or FOLFIRI chemotherapy in combination with anti-VEGF therapy (e.g., bevacizumab). Inhibitors for specific RAS mutations (e.g., sotorasib and adagrasib for KRAS^G12C^) are in clinical development. About 5% of mCRC expresses BRAF^V600E^, and recently, the combination of encorafenib+anti-EGFR (cetuximab) was FDA-approved for front-line treatment. *HER2* is amplified in ~5% of mCRC, and a preferred front-line treatment is trastuzumab+lapatinib. For RAS^WT^ mCRC without *BRAF* mutation or *HER2* amplification, chemotherapy combined with anti-EGFR therapy (cetuximab or panitumumab) is recommended front-line treatment. Acquired resistance to anti-EGFR therapy can result from *ALK*-fusion or *NTRK*-fusion with the indicated treatment.

Following initiating APC mutations, CRC tumors developing via the CIN pathway frequently include activating mutations in *KRAS* (~40%) and *NRAS* (3–5%) [21]. Activating mutations in RAS-family GTPases or in *BRAF* decouple proliferative signaling via the RAS/RAF/MEK pathway from upstream signaling regulated by EGFR and other receptor tyrosine kinases (RTKs) leading to uncontrolled cell growth. EGFR plays an important role in normal colon development [22], and it is an important target for treatment of mCRC in which the RAS/RAF/MEK pathway is not activated through mutation (Figure 1). Molecular testing for activating *KRAS* and *NRAS* mutations (comprehensive RAS panel) and *BRAF* mutations is recommended by the National Comprehensive Cancer Network (NCCN) and the European Society for Medical Oncology (ESMO) to select patients for anti-EGFR therapy [23]. Activating mutations in *KRAS* may also contribute to an immunosuppressive tumor microenvironment [24] and the lack of response of mCRC originating via the CIN pathway rather than the MSI pathway. Direct pharmacological targeting of mutated *KRAS* was not possible until recently, and drugs for KRAS mutations common in mCRC (e.g., KRAS^G12D^) are not yet clinically validated. Hence, cytotoxic chemotherapy using fluoropyrimidine drugs (FPs), 5-fluororuracil (5-FU), or capecitabine in regimens such as FOLFOX and FOLFIRI is, in many cases, a preferred option for front-line treatment of mCRC with activating *KRAS* mutations.

## 3. Targeting EGFR in mCRC

Epidermal growth factor (EGF) receptor (EGFR/ErbB1/HER1) is widely expressed in CRC, and its elevated expression is associated with metastases and poor outcomes [25]. EGFR signaling proceeds via the binding of EGF or another EGFR-specific ligand (TGF-α, amphiregulin (AREG)) followed by EGFR-dimer formation, which promotes transautophosphorylation [26]. The EGFR family includes HER2/Neu/ERBB2, HER3/ERBB3, and HER4/ERBB4 in addition to EGFR/ErbB1/HER1. The formation of either homo- or heterodimers is possible resulting in 28 possible combinations that regulate diverse signaling pathways including RAS/RAF/MAPK, PI3K/AKT/mTOR, and JAK/STAT3 that collectively promote mCRC progression. HER3 and HER4 bind neuregulins (NRGs), and ligand binding promotes the formation of homodimers or preferentially heterodimers with HER2. HER2/HER3 and HER3/HER4 heterodimers are tumor promoting [27].

### 3.1. Targeting EGFR

**Figure 2:**
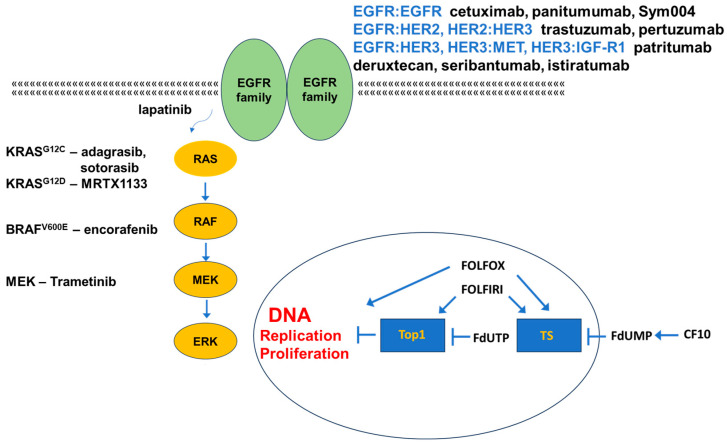
Therapeutic targeting of mCRC. EGFR-family signaling initiates proliferative signaling in mCRC via the RAS/RAF/MAPK cascade. Monoclonal antibodies targeting EGFR dimer and specific heterodimers have activity in mCRC without mutations in a RAS protein or BRAF. Small molecule inhibitors of specific KRAS mutant forms and BRAF^V600E^ have activity in mCRC. Fluoropyrimidine-based chemotherapy targets thymidylate synthase (TS) and DNA topoisomerase 1 (Top1) and causes DNA damage and replication stress.

The liver is the most frequent site of CRC metastasis, and 79% of CRC liver metastases express EGFR (vs. 38% non-metastatic CRC); strategies targeting EGFR are central to the treatment of liver metastatic disease (Figure 2). EGFR is frequently overexpressed but is rarely mutated in CRC, and tyrosine kinase inhibitors (TKIs) targeting mutant-selective EGFR are not effective as single agents for mCRC treatment [28]. However, two anti-EGFR monoclonal antibodies (mAbs) are widely used for mCRC treatment: (i) cetuximab, a chimeric human–mouse mAb; and (ii) panitumumab, a fully humanized mAb. Anti-EGFR mAbs are used in combination with fluoropyrimidine-based combination chemotherapy regimens (FOLFOX and FOLFIRI) for front-line treatment of mCRC provided that there are no activating *RAS* or *BRAF* mutations downstream of EGFR that would render anti-EGFR therapy ineffective. Elevated plasma levels of EGFR ligands such as epiregulin (EREG) and amphiregulin (AREG) may be indicative of resistance to anti-EGFR therapy [29]. Cetuximab and panitumumab treatment results in similar survival benefit. Although not common, the use of these mAbs may be associated with adverse reactions with decreased paronychia (nail infection) observed with panitumumab [30].

### 3.2. Targeting Other EGFR-Family Members

Increasingly, the expression of EGFR-family members other than EGFR/ErbB1/HER1 is used to guide treatment. HER2 is overexpressed in 3–5% of mCRC [31], and *HER2* amplification is associated with lower incidence of activating *KRAS* mutations and occurs more commonly in left-sided CRC tumors. HER2 overexpression is a negative predictor associated with reduced efficacy of anti-EGFR therapy [31], and >36% of cetuximab-resistant mCRC with wild-type KRAS, NRAS, BRAF, and PI3K was found to display HER2 overexpression [32]. The combined inhibition of EGFR and HER2 displayed promising activity in mCRC patients with HER2 overexpression; however, monotherapy with the anti-HER2 mAb trastuzumab shows relatively low efficacy, although combining trastuzumab with chemotherapy displays promise. Lapatanib [33], a small molecule inhibitor of EGFR and HER2, also shows promising activity in combination with trastuzumab for the treatment of HER2-overexpressed mCRC [34]. Combining pertuzumab, a mAb that prevents HER2/HER3 dimer formation, with trastuzumab also shows promise in combination for this indication [35]. The National Comprehensive Cancer Network recommends the systematic evaluation of HER2 status in determining mCRC treatment, and multiple new approaches to target HER2 overexpression in mCRC are in clinical development [36].

HER3 is widely expressed in mCRC, and elevated HER3 expression was shown to be an independent prognostic factor that was associated with poor outcomes [37]. HER3 displays intrinsically low kinase activity and must dimerize with either another EGFR-family member, including HER2 and EGFR, or a non-EGFR receptor tyrosine kinase (RTKs), including fibroblast growth factor receptor (FGFR), MET, or Axl to stimulate cell survival and proliferation signaling [38]. HER3 was also shown to be upregulated in CRC cells in response to factors secreted from liver endothelial cells (ECs), and blocking HER3 using a humanized anti-HER3 mAb seribantumab inhibited EC-induced CRC survival [39]. Multiple antibody therapies are in development targeting HER3 [40] including patritumab deruxtecan that includes an anti-HER3 mAb linked to a topoisomerase 1 (Top1) inhibitor payload [41]. Multi-targeting antibodies that include HER3 as a target such as istiratumab, which binds HER3 and insulin-like growth factor 1 receptor (IGF-1R) [42], also display promise for CRC. HER4 is also expressed in mCRC, and, paradoxically, it may act as either a tumor suppressor or an oncoprotein depending on its dimerization status [43]. In contrast to other EGFR-family members, relatively little therapeutic development for mCRC is directly targeted at HER4.

### 3.3. Alternative Targets in EGFR-Resistant mCRC

First-line treatment of mCRC includes anti-EGFR antibodies (cetuximab or panitumumab) in combination with FP-based chemotherapy unless activating mutations in RAS proteins or BRAF confer intrinsic resistance. Elevated levels of EGF and other EGFR ligands (e.g., TGF-α, epiregulin (EREG), and AREG) also are indicative of decreased probability of anti-EGFR efficacy [44]. Progressive disease following anti-EGFR therapy may result from acquired resistance [45,46], which may result from any one of several causes [47]. Several somatic mutations in an EGFR extracellular domain (ECD), including G465R, G465E, S468R, and S492R, interfere with the binding of anti-EGFR antibodies to EGFR. Post-translational modifications in an extracellular domain, such as arginine methylation (R198/R200) can also inhibit binding of EGFR by anti-EGFR antibodies. The S492R mutation is selective for resistance to cetuximab, and treatment with panitumumab may be an option for patients with this EGFR mutation [48]. Monoclonal antibodies that bind different epitopes of EGFR, including Sym004 [49], which is a combination of two mAbs that target EGFR extracellular domain III, and MM-151 [50], have the potential to overcome these forms of acquired resistance to cetuximab or panitumumab.

Acquired resistance to anti-EGFR therapy can also result from the activation of key signaling pathways downstream of EGFR, either through acquired activating mutations in the RAS/RAF/MEK or PI3K/AKT/mTOR pathways or through amplification or activation of alternative receptor tyrosine kinases capable of alternatively activating these signaling cascades independently of EGFR, including MET and IGF-1R. MET amplification, which is rare in untreated mCRC (0.6%), is detected in ~7% of mCRC patients treated with anti-EGFR therapy. The combination of anti-MET therapy using a multi-tyrosine kinase inhibitor cabozantinib [51] or tepotinib [52] with anti-EGFR therapy may be effective in patients with acquired resistance to anti-EGFR therapy through MET amplification [53] (Figure 1). Proliferative and cell survival signaling via RAS/RAF/MEK and PI3K/AKT/mTOR may also be upregulated downstream of IGF-1R, potentially circumventing a requirement for EGFR-mediated signaling and attenuating the effect of anti-EGFR therapy. Studies combining cetuximab with anti-IGF-1R therapies [54,55] have not yet yielded favorable clinical outcomes that would be consistent with this approach overcoming acquired resistance to anti-EGFR therapy. Regorafenib, a multi-kinase inhibitor targeting EGFR1-3, TIE2 (angiopoietin receptor), platelet-derived growth factor receptor β (PDGFR-β), FGFR, KIT, RET, and BRAF [56], is approved for the treatment of refractory mCRC [57] and, in combination with cetuximab, displays potential for overcoming acquired resistance to anti-EGFR therapy [58].

The development of acquired resistance to anti-EGFR therapy can result from acquired mutations in downstream signaling pathways (RAS/RAF/MAPK and PI3K/AKT/mTOR) with one study finding that 28.6% of patients showed activating alterations in either a RAS protein, BRAF, ERK, or an alternative receptor tyrosine kinase receptor (RTK) [59]. Combining anti-EGFR therapy with a MEK inhibitor (e.g., selumetinib [60] or pimasertib [61]) may hold therapeutic promise in limiting resistance via this mechanism. Resistance to anti-EGFR therapy also occurs in mCRC expressing *ALK* fusions (0.2% of cases) or neurotrophic tyrosine receptor kinase (*NTRK)* fusions (0.7%), and these may be detected through sequencing circulating tumor DNA (ctDNA) [62]. Tumors expressing the fusion gene protein products benefit from treatment with ALK inhibitors such as ceritinib [63] or NTRK inhibitors such as larotrectinib or entrectinib [64].

## 4. Targeting Mutant KRAS

Mutations in *KRAS* frequently occur in mCRC, at ~40%, while an additional 4% display activating *NRAS* mutation with few cases displaying *HRAS* mutations. Determining the *RAS* mutation status is central to identifying an appropriate therapeutic strategy for mCRC patients since activating mutations in any *RAS* gene or in *BRAF* render therapeutic targeting of EGFR and EGFR-family members ineffective. RAS is activated downstream of EGFR transphosphorylation via a signaling cascade composed of Shc2 → GRB2 → SOS (son of sevenless). SOS is a guanosine exchange factor (GEF) that stimulates the exchange of GDP for GTP on RAS converting it to its active state. Activating *RAS* mutations bypass a requirement for EGFR-pathway activation to stimulate RAS-mediated signaling and maintain RAS in a constitutively activated state. In its activated state, RAS stimulates cell proliferation and survival signaling by activating the RAF/MEK/ERK, PI3K/AKT/mTOR, and Ral pathways.

Despite the prevalence of activating *KRAS* mutation in CRC and its importance for determining the choice of therapy in mCRC [65], until recently, no drugs were available to directly target mutant KRAS. The most frequent *KRAS* mutations in CRC occur at codon 12 (75%), while activating mutations at codon 13 (23%) and codon 61 (2%) also occur [66]. In general, activating *KRAS* mutations in mCRC are associated with accelerated metastatic progression [67] and relatively poor outcomes with G12S mutations associated with a particularly high mortality rate [68]. Challenges in targeting KRAS through direct targeting of the GTP binding pocket include the high affinity for its native substrate GTP, which is abundant in cancer cells [69]. Alternative strategies, including inhibiting post-translational modification of KRAS using farnesyl transferase inhibitors (FTI [70], e.g., tipifarnib [71] and salirasib [72]) to prevent localization to the inner plasma membrane, did not, however, evoke objective responses in clinical trials, possibly due to activation of a bypass prenylation pathway via geranylgeranylation [73]. Alternative therapeutic approaches include inhibiting the interaction of farnesylated KRAS with cGMP phosphodiesterase delta (PDEδ) using small molecule inhibitors including deltarasin; however, this approach is not selective for cancer cells and causes systemic toxicities [74]. Targeting mutant *KRAS* at the mRNA or gene level is also under investigation; a 16mer antisense oligonucleotide (AZD4785) targeting the 3′-untranslated region was evaluated in a Phase 1 clinical trial, but no follow-up studies are yet reported [75].

Studies in recent years have, for the first time, demonstrated the feasibility and clinical utility of directly targeting mutant KRAS with small molecule therapeutics designed to exploit specific structural features present only in the mutant protein [76] (Table 1). Therapeutic development has primarily focused on targeting *KRAS* mutations at codon 12 with compounds targeting G12C activating mutations most advanced with some development of G12D and G12V specific therapies. In contrast to lung adenocarcinoma, in which it is present in 11% of cases [77], KRAS^G12C^ occurs in <4% of mCRC cases [78]. Therapeutic development targeting KRAS^G12C^ has progressed in mCRC, and clinical development of RAS direct inhibitors has begun to impact treatment [79]. In a Phase 2 study, Sotorasib (AMG510, Amgen), a selective KRAS^G12C^ inhibitor, displayed activity in mCRC patients expressing the target mutation with approximately 10% of patients displaying an objective response [80]. Similar results were obtained with the KRAS^G12C^-targeted therapy adagrasib (MRTX849, Mirati) in clinical studies for mCRC [81]. To circumvent the relatively modest response rates in mCRC patients observed with selective KRAS^G12C^ inhibitors as single agents, combination therapy with anti-EGFR antibodies is under investigation with promising results. The rationale for combining selective KRAS^MT^ inhibition therapy with anti-EGFR therapy is that adaptive feedback mediated by EGFR may stimulate the RAS/MAPK pathway [82]. Sotorasib and adagrasib both displayed increased activity in combination with panitumumab [83] (CODEBREAK 300, NCT05198934) and cetuximab [84] (KRYSTAL-1, NCT03785249), respectively. KRAS^G12C^ inhibitors are also being investigated in combination with MEK inhibitors, anti-AKT drugs, and other strategies to block feedback and bypass pathways that alternatively activate proliferative and survival signaling [79]. Additional KRAS^G12C^ inhibitors are undergoing clinical development including JNJ-74699157 (NCT04006301) and LY3499446 (NCT04165031).

KRAS^G12D^ mutation is relatively more frequent than KRAS^G12C^ in mCRC, particularly in black patients (13% vs. 6% in white patients) [4]. However, the development of small molecule inhibitors selective for this mutation has lagged behind the development of KRAS^G12C^ inhibitors. Recent studies with the KRAS^G12D^ specific inhibitor MRTX1133 [85] have, however, showed promise that this mutant form of *KRAS* is also druggable. MRTX1133 displayed strong and selective activity in KRAS^G12D^ xenograft models; however, pERK reactivation is detected with continued treatment indicating that co-development with inhibitors of bypass pathways may be important for clinical development. Multiple studies have demonstrated the clinical potential of targeting downstream signaling to selectively intervene in *KRAS*-mutant cancer. Encouraging results were obtained in clinical studies for non-small cell lung cancer (NSCLC) with MEK inhibitors including trametinib; however, in CRC, it was observed that a MEK inhibitor, AZD6244, caused the upregulation of c-MET/JAK/STAT signaling as an adaptive resistance mechanism. Further development combining MEK and MET inhibition [86] is being further evaluated in clinical studies (NCT02510001) to target both downstream signaling and putative adaptive resistance for the treatment of *KRAS*-mutant CRC. The challenges of targeting mutant *KRAS* and the insufficiency of MEK inhibition led to studies investigating complementary pathways to potentially enhance MEK inhibition resulting in the identification of Src inhibition with dasatinib as a potential effective combination treatment; but in vivo studies have not yet been successful with this approach [87].

## 5. Targeting Mutant BRAF

*BRAF* mutations occur in ~8.5% of CRC cases [88] with BRAF^V600E^, a class I BRAF mutation and the predominant mutation comprising two-thirds of all BRAF mutations in CRC. Class I BRAF mutations enable BRAF to signal as a monomer and independent of upstream input or RAS activation [89]. Class II mutations that, in CRC, include BRAF^K601E^ and BRAF kinase fusions are also independent of upstream activation but function as dimers. Front-line chemotherapy for BRAF^V600E^ mCRC is not firmly established but frequently consists of doublet therapy (FOLFOX or FOLFIRI) in combination with targeted therapy (e.g., bevacizumab) [90]. BRAF^V600E^ mutation is associated with decreased median survival, only 13.5 months relative to 30.6 months for *BRAF* wild-type tumors [4], while non-V600E BRAF mutations did not serve as a factor for poor prognosis in mCRC [91]. The increased aggressiveness of BRAF^V600E^ mCRC led to evaluation of more aggressive first-line chemotherapy; however, triplet therapy with FOLFOXIRI did not demonstrate an advantage relative to doublet therapy (FOLFOX or FOLFIRI) with bevacizumab [92]. Consistent with BRAF^V600E^ mutation, signaling independent of upstream input, anti-EGFR therapy is not useful in patients with BRAF^V600E^ tumors as single agents [93] or in combination with chemotherapy [94]. Class II *BRAF* mutations also display limited response to anti-EGFR therapy, while class III BRAF mutations, which are dependent on upstream input for signaling, are responsive to anti-EGFR therapy [95]. Inhibition of BRAF^V600E^ with vemurafenib [96] or dabrafenib [97] has limited activity in mCRC even when combined with anti-MEK therapy with trametinib. Feedback upregulation of EGFR is considered the cause for the lack of efficacy for drugs that directly target mutant BRAF^V600E^ to be ineffective in mCRC with this activating mutation, and acquired resistance to anti-BRAF^V600E^ therapy is associated with EGFR mutations and amplification and activating *RAS* or alternative *BRAF* mutations [98]. Inclusion of anti-EGFR therapy (e.g., cetuximab) in combination with direct targeting of BRAF^V600E^ (encorafenib) increased the objective response rate to 19.5% relative to control (irinotecan or FOLFIRI plus cetuximab; 1.8%) [99], and inclusion of anti-MEK therapy (binimetinib) further increased the objective response rate to 26.8%. Doublet therapy (encorafenib+cetuximab) is FDA-approved as the standard of care for BRAF^V600E^ mutant mCRC [89]. Activation of the WNT/β-catenin pathway may also cause resistance to anti-BRAF therapy, and the combination of a WNT inhibitor (WNT974) with encorafenib and cetuximab is under evaluation for the treatment of BRAF^V600E^-mutant CRC (NCT02278133). Amplification of cyclin D1 and mutation in CDK4/6 and other CDKs are associated with resistance to BRAF inhibitors [100], and inclusion of CDK inhibitors, including CDK12 [101], which is activated via the RAS/MAPK pathway, with anti-BRAF therapy is being investigated for overcoming resistance to targeted therapy in multiple types of cancer with BRAF^V600E^ mutations.

## 6. Alternative Targets of Activated EGFR

Much of the current success in targeted therapy for mCRC is based on anti-EGFR antibody therapy and, more recently, anti-KRAS and anti-BRAF therapies. In addition to activating the RAS/RAF/MAPK pathway, EGFR signaling may activate the PI3K/AKT/mTOR and RAL pathways to stimulate proliferative and survival signaling. Mutations in the PI3K/AKT/mTOR pathway are common in mCRC [102], contribute to malignant transformation [103], and are implicated in resistance to front-line therapy [104]. However, targeting of the PI3K/AKT/mTOR pathway has not yet proven to be a successful strategy for treating mCRC [105], although this remains an area of investigation. RALA and RALB are upregulated in CRC [106] and are implicated in invasion and metastasis in pancreatic cancer, but analogous roles in CRC have not been reported [107]. RAL proteins activate EGFR/MAPK signaling [108] in the intestine in Drosophila, but a role in human CRC development is not established. However, since RAL proteins have a role as RAS effectors, the potential of this pathway to be activated in acquired resistance may emerge.

## 7. Wnt-Pathway Targeting

Wnt signaling plays a major role in sustaining the dynamic biology of intestinal epithelia in which proliferative progenitors derived from stem cells at the base of the crypts propagate culminating in differentiation into one of the mature cell lineages present in villi: goblet cells, enteroendocrine cells, and absorptive epithelial cells. The Wnt/β-catenin pathway also plays a critical role in maintaining the cancer stem cell (CSC) population in malignant tissues making Wnt a potential target in CRC [109], even though its essential role in normal intestine function makes toxicity of agents targeting the Wnt pathway a serious concern. While loss of the tumor suppressor APC is the characteristic mutation resulting in Wnt-pathway activation in CRC etiology, cancer therapeutic development targeting the Wnt pathway is focused on downstream mediators including tumor necrosis factor receptor-associated factor (TRAF) and NCK-interacting protein kinase (TNIK), an essential regulator of the TCF4/β-catenin complex [110]. The TNIK inhibitor NCB0846 as well as the FDA-approved mebendazole that targets TNIK display promising activity in preclinical CRC models demonstrating the therapeutic potential of Wnt-pathway targeting for CRC treatment [111]. A Tankyrase inhibitor, IWR-1 [112], also shows promise in targeting CSCs by modulating PARylation of AXIN, a key component of both the β-catenin destruction complex and the WNT signalosome [113]. Other modulators of canonical Wnt signaling include niclosamide that decreases Dv1-2 and β-catenin expression [114] and now is in clinical trials for CRC [115]. Recently, niclosamide and two other β-catenin inhibitors (FH535 [116] and LF3 [117]) have been shown to downregulate MACC1-regulated S100A4 expression to decrease CRC propensity for metastatic progression [118]. Multiple agents targeting both canonical and the β-catenin-independent non-canonical Wnt signaling including v-ATPase inhibitors and anti-RSPO antibody are undergoing clinical trials in CRC and other malignancies [119,120].

## 8. Targeting VEGFR

The anti-VEGF antibody bevacizumab (BEV) is used in combination with chemotherapy (FOLFOX or FOLFIRI) for the treatment of mCRC with activating mutations in the RAS/RAF/MAPK pathway [121]. Multiple clinical trials support an advantage for anti-VEGF therapy in combination with chemotherapy in second-line mCRC [122]. A rationale for combining anti-VEGF therapy with chemotherapy is that anti-VEGF therapy caused tumor vasculature to normalize resulting in increased drug delivery to tumors [123]. However, in some studies, BEV actually decreased tumor perfusion and drug delivery [124]. Angiogenesis is an important process promoting mCRC progression; and, in addition to BEV, which targets VEGF-A, several drugs targeting angiogenesis display activity in mCRC, including ramucirumab, a humanized mAb targeting VEGF-R2; regorafenib, which inhibits multiple RTKs; and aflibercept, a recombinant fusion protein that serves as a decoy receptor for VEGF-A, VEGF-B, and placental growth factor [125]. In front-line treatment for mCRC, anti-VEGF therapy was shown to be comparable with anti-EGFR therapy when either was combined with chemotherapy [126], and while some previous studies indicated that chemotherapy was more effective in right-sided CRC [127], no side dependence was detected. Second-line therapy may involve an alternative anti-VEGF drug (e.g., chemotherapy+Aflibercept) for patients progressing following treatment with front-line chemotherapy+BEV [125], while regorafenib whose targets include VEGF-R1, VEGF-R2, and VEGF-R3 or Fruquintinib is used in combination with TAS-102 (combination of trifluridine and Tiperacil) for third-line treatment [128].

## 9. FP-Based Chemotherapy in CRC

5-FU-based chemotherapy is central to both adjuvant therapy of primary CRC and for systemic therapy for mCRC. However, deficiencies in DNA mismatch repair (dMMR or microsatellite high (MSI-high)) render FP-based chemotherapy relatively ineffective. Approximately 15% of primary CRC is dMMR/MSI-high, but in mCRC, this is only <5% of cases. MSI-high or dMMR mCRC patients favorably respond to immune checkpoint blockade (ICB), and this is now the preferred front-line treatment [129]. Microsatellite instability involves mutations in DNA mismatch repair (MMR) genes (MLH1, MSH2, MSH6, and PMS2) or epigenetic silencing of MLH1. About 3% of all CRC diagnoses result from germline mutation in one of the MMR genes (e.g., Lynch syndrome). Deficiencies in MMR are detected using a consensus marker panel (Bethesda panel) and immunohistochemistry (IHC), although a more rapid PCR method using seven markers of MSI (Idylla MSI assay) appears promising [130].

### 9.1. Adjuvant Chemotherapy in Primary Colorectal Cancer

Adjuvant chemotherapy is administered to reduce the risk for disease recurrence in CRC patients undergoing surgical resection for primary colon cancer with curative intent [131]. Adjuvant chemotherapy is recommended for most CRC patients with stage III CRC and for stage II CRC patients who are deemed at high risk for disease recurrence. Factors associated with high risk for stage IIA (T3) disease include sampling <12 lymph nodes in the surgical specimen, an undifferentiated tumor grade, and perineural or lymphovascular invasion [132]. For CRC patients with stage IIB disease (T4, lesions penetrating visceral peritoneum) and stage IIC CRC (T4, invasion of surrounding organs), adjuvant chemotherapy is recommended. The inclusion of oxaliplatin with fluoropyrimidine chemotherapy (e.g., 5-FU or capecitabine with leucovorin (LV)) provides benefit relative to adjuvant FP chemotherapy alone. However, oxaliplatin chemotherapy is associated with peripheral neuropathy, and strategies to limit the duration of oxaliplatin treatment in the adjuvant setting are being investigated to reduce toxicity without compromising efficacy [133]. Circulating tumor DNA (ctDNA) is emerging as a predictive factor for the necessity of adjuvant chemotherapy, and the use of ctDNA is expected to inform standard practice in the near future, potentially reducing toxicity due to unnecessary chemotherapy [134,135].

### 9.2. Chemotherapy in Metastatic Colorectal Cancer

**Figure 3:**
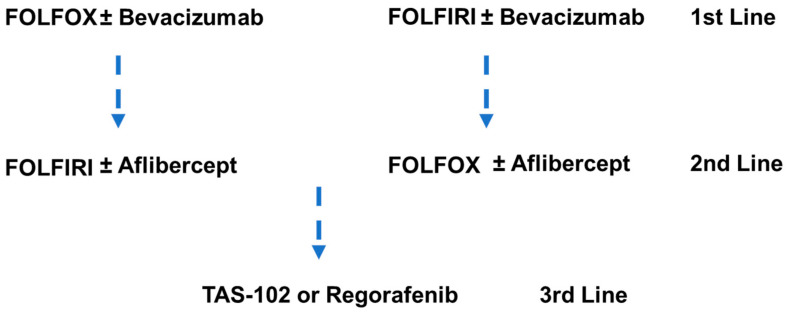
Sequential administration of FP-based chemotherapy for the treatment of mCRC that is RAS^MT^ and microsatellite-stable (MSS) following disease progression. Front-line treatment consists of either FOLFOX or FOLFIRI, which may be combined with anti-VEGF therapy. Second-line treatment generally uses the alternative FP-based regimen (e.g., FOLFOX → FOLFIRI) and may be combined with an alternative anti-VEGF agent (e.g., Aflibercept). Third-line treatment uses an alternative FP-based treatment, TAS-102, in which FP trifluridine is combined with Tiperacil. Regorafenib is also widely used in third-line treatment of mCRC.

5-FU-based chemotherapy is central to systemic therapy for mCRC [136] (Figure 3). The introduction of 5-FU and its modulation with leucovorin (LV) resulted in a substantial benefit in overall survival, 3–6 months [137]. Capecitabine, an oral FP that is enzymatically converted to 5-FU, was shown to be equivalent to 5-FU in survival benefit [138]. FP-based doublet therapies in which 5-FU/LV is combined with oxaliplatin (FOLFOX) or irinotecan (FOLFIRI) are now a preferred first-line treatment for mCRC. Addition of oxaliplatin to 5-FU/LV resulted in a higher response rate (50.7 vs. 22.3%) and improved progression-free survival (9.0 vs. 6.2 months), although overall survival (16.2 vs. 14.7 months) was not significantly improved [139]. Similar trends were observed for the addition of irinotecan to 5-FU/LV that resulted in an improved objective response rate (49 vs. 31%) and longer time to progression (6.7 vs. 4.4 months), but, in this case, overall survival benefit was significantly increased (17.4 vs. 14.1 months) [140].

FP-based chemotherapy, either FOLFOX/XELOX or FOLFIRI/XELIRI, is widely used in the front-line treatment of mCRC, often in combination with anti-EGFR or anti-VEGF therapy depending on the patient mutation profile (Figure 1). Multiple FOLFOX variants are clinically used. For example, FOLFOX4 (oxaliplatin 85 mg/m^2^ on day 1; LV 200 mg/m^2^, bolus 5-FU 400 mg/m^2^, and continuous infusion 5-FU 600 mg/m^2^ over 22 h, days 1–2 every two weeks) resulted in an overall response rate (ORR) of 23.5% and progression-free survival (PFS) of 5.1 months. The alternative FP doublet therapy is often used for second-line treatment following disease progression (e.g., FOLFOX (1st) → FOLFIRI (2nd)), and there is no evidence for a preferred sequencing of these regimens [141]. The overall percent of mCRC patients treated with FOLFOX or FOLFIRI has not diminished even as the introduction of targeted therapies since 2012 has improved OS [11]. The duration of chemotherapy in first-line therapy is also a matter of debate as serious adverse events including peripheral neuropathy are associated with the oxaliplatin component in FOLFOX. Collectively, the implementation of targeted therapies directed at actionable driver mutations in conjunction with strategies to counter feedback inhibition and circumvent compensatory signaling pathways is extending survival in mCRC patients with some of the historically most rapidly lethal mCRC sub-types (e.g., BRAF^V600E^).

Chemotherapy is important for reducing tumor burden prior to resection and qualifying patients with potentially resectable disease for surgery, which significantly improves the likelihood of long-term survival [16]. For patients deemed able to tolerate more aggressive therapy, the inclusion of both irinotecan and oxaliplatin together with 5-FU/LV in FOLFOXFIRI triplet chemotherapy improved ORR, PFS, and median OS relative to doublet therapy [142]. Furthermore, an improved surgical resection rate was found with triplet therapy [143]. In the third-line setting, a thymidine-based FP analog trifluridine is used rather than 5-FU. TAS-102, a combination of trifluridine and a thymidine phosphorylase inhibitor (Tiperacil), was shown to improve OS relative to placebo [144]. TAS-102 displays promising activity in combination with bevacizumab for the third-line treatment of mCRC. The therapeutic benefit of TAS-102 in the third-line setting for mCRC is consistent with FPs with more DNA-directed mechanism having therapeutic potential, and the second-generation polymeric FP CF10 shows promising activity in preclinical studies [145].

## 10. Immunotherapy Approaches for mCRC

Immune checkpoint blockade (ICB) therapy with anti-PD-1 drug Pembrolizumab or Nivolumab is the approved first-line treatment for mCRC that is dMMR or MSI-high; however, this is only <5% of mCRC, and some estimates indicate that only 2% of mCRC patients are candidates for successful therapy with ICB therapy [129]. The lack of efficacy for ICB therapy in MSS mCRC is considered to result from a relatively lower tumor mutational burden (TMB) reducing the presentation of neo-antigens. mCRC that originates via Pol epsilon mutations displays relatively higher TMB, and these patients show increased response to ICB therapy [146]. Published genomic studies show that 7.5% of mCRC patients with MSS tumor display high TMB, and sequencing may identify candidates for ICB therapy not detected based on dMMR/MSI [147]. Clinical studies have investigated the potential of chemotherapy to improve the activity of immunotherapy in MSS mCRC, but no therapeutic benefit has been established for this approach [148].

## 11. Conclusions and Future Perspectives

Improvements in precision therapy for mCRC have yielded impressive gains in survival with median overall survival (OS) for mCRC now exceeding 32 months [11] and many patients now surviving for third-line treatment. A limitation is that reported improvements in survival are often based on outcomes from clinical trials that involve a small percent of patients and often those with favorable performance characteristics. Consistent with this, 5-year survival rates in mCRC remain dismal [6] indicating a great need for further therapeutic advances. For decades, targeted anti-EGFR therapy has proven highly successful in treating mCRC for which neither activating *RAS* or *BRAF* mutations were detected [30]. Recent progress in accounting for the contribution of other EGFR-family members, HER3 and especially HER2, as drivers of malignancy progression and drug resistance is enabling more precise targeting of this important signaling pathway. The role of alternative RTKs in compensatory signaling with EGFR inhibition, or as driver mutations when expressed as an oncogenic fusion protein, is established thus allowing for precision targeting to be expanded to a more comprehensive list of mCRC-related targets. The direct targeting of specific *KRAS* mutations with small molecule inhibitors is starting to impact mCRC treatment and will likely continue to expand as alternative *KRAS* mutations that are more common in mCRC become druggable targets and as strategies to control compensatory signaling pathways that maintain cell viability and proliferative potential upon KRAS^MUT^ inhibition become clinically validated [84].

While the development of targeted therapies directed at RTKs and KRAS^MUT^ and BRAF^V600E^ inhibitors is extensively contributing to improved survival in mCRC, the majority of patients still receive FP-based chemotherapy, and this is expected to continue even as new targeted agents enter clinical development. The refinement of FP-based chemotherapy regimens and their sequential administration to overcome resistance has resulted in important advances in survival. The more recent introduction of TAS-102 (trifluridine-tipiracil) [149] is further extending the survival of patients in third-line treatment. The long-established role for FP chemotherapy in CRC treatment holds promise for new FPs, including CF10, a polymeric fluoropyrimidine [145], to further advance the survival benefit of this important drug class in mCRC.

There is also potential to identify novel targets that are important for mCRC progression. The increased availability and reduced cost of next-generation sequencing technologies may enable important new targets to be identified [150]. Furthermore, there remains the potential to selectively target proteins and pathways with established importance in mCRC progression including TGF-β, p53, and PI3K/AKT/mTOR in new ways such as targeting regulatory miRNA, enabling therapeutic targeting for mCRC for established targets that has not proven possible to date. Collectively, these approaches may further extend survival and eventually render mCRC manageable long-term chemotherapy in conjunction with surgery and other modalities in conjunction with surgery and radiation.

## Figures and Tables

**Table 1:** Anti-Ras therapies approved and in clinical development.

Drug	Brand Name	Company	Mutation	Status
Sotorasib	Lumakras	AMGEN	G12C	Approved
Adagrasib	KRAZATI	Mirati	G12C	Approved
JDQ443		Novartis	G12C	Phase 2
MRTX1133		Mirati	G12D	Phase 1
RMC-6236		Revolution	Multiple	Phase 1

## Data Availability

The data can be shared upon request.
